# A New Method for Immobilization of His-Tagged Proteins with the Application of Low-Frequency AC Electric Field

**DOI:** 10.3390/s18030784

**Published:** 2018-03-05

**Authors:** Shunsuke Takahashi, Kazuki Kishi, Ryota Hiraga, Kazuki Hayashi, Youhei Mamada, Masahiko Oshige, Shinji Katsura

**Affiliations:** Department of Environmental Engineering Science, Graduate School of Science and Technology, Gunma University, Kiryu, Gunma 376-8515, Japan; t14807002@gunma-u.ac.jp (S.T.); t15803019@gunma-u.ac.jp (K.K.); t12304030@gunma-u.ac.jp (R.H.); t14803043@gunma-u.ac.jp (K.H.); t14303090@gunma-u.ac.jp (Y.M.); oshige@gunma-u.ac.jp (M.O.)

**Keywords:** protein immobilization, mass transport phenomenon, self-assembled monolayer, surface treatment, low-frequency alternating current (AC) electric field

## Abstract

Continued advancement of protein array, bioelectrode, and biosensor technologies is necessary to develop methods for higher amount and highly oriented immobilization activity of proteins. In pursuit of these goals, we developed a new immobilization method by combining electrostatic transport and subsequent molecular diffusion of protein molecules. Our developed immobilization method is based on a model that transports proteins toward the substrate surface due to steep concentration gradient generated by low-frequency AC electric field. The immobilization of the maximum amounts can be obtained by the application of the AC voltage of 80 Vpp, 20 Hz both for His-tagged Green Fluorescent Protein (GFP) and *Discosoma* sp. Red Fluorescent Protein (DsRed), used as model proteins. The amounts of the immobilized His-tagged GFP and DsRed were approximately seven-fold higher than that in the absence of the application of low-frequency AC electric field. Furthermore, the positively and negatively charged His-tagged GFP at acidic and alkaline pH were immobilized by applying of low-frequency AC electric field, whereas the non-charged His-tagged GFP at the pH corresponding to its isoelectric point (pI) was not immobilized. Therefore, unless the pH is equal to pI, the immobilization of electrically charged proteins was strongly enhanced through electrostatic transport and subsequent molecular diffusion.

## 1. Introduction

The immobilization of proteins on the surface of solid materials is a key technology in the production of protein arrays, biosensors, and bioelectrodes for use in the analytical and bioelectronics fields. For these applications, it also is necessary to achieve higher amounts of immobilized proteins, but also to control the orientation of the immobilized proteins in order to keep those activities [1,2]. A number of different methods have been reported for immobilizing proteins on a variety of surfaces [3,4,5,6]. Proteins may be immobilized by physical absorption (e.g., dot blot [7,8] and polyacrylamide gel [9,10] methods), through covalent coupling or cross-linking [3,11,12,13,14,15], as a self-assembled monolayer (SAM) [16,17,18,19], or through affinity interactions (e.g., avidin/biotin [3,20,21], nickel-nitrilotriacetic acid (Ni-NTA)/His-tagged protein [3,22,23,24], and intein methods [1,25,26].

These protein physical absorption/immobilization techniques were designed for immobilizing large amounts of highly active proteins at a high density. However, there is still a need for techniques to maintain protein activities during the immobilization processes. Immobilizing large amounts of proteins of interest over a minimal surface area remains challenging for many applications, as does maintaining the orientation of immobilized proteins so as to maintain their activity. To solve the problem of maintaining the proper orientation of immobilized proteins, we have developed protein immobilization methods [27,28] based on specific binding between NTA and the His-tagged protein that was developed by Hochuli et al. [29]. However, most methods including our previously developed methods were immobilized by spotting the purified protein solution. Because it is difficult to apply the operation of stirring and shaking to such small volumes of the spotted protein solution for those immobilization methods, transport of proteins toward the substrate is limited by molecule diffusion and a long time is required for the immobilization. Thus, it is necessary to maintain the spotted solution in the position for a long time without evaporating. For this reason, higher amounts of proteins cannot be immobilized because of the rate-limit of transport. Mass transport of proteins is principally driven by molecular diffusion, whereas proteins are higher in molecular weight, resulting smaller diffusion coefficient. Therefore, transportation toward the substrates is quite insufficient for preparation of protein arrays, biosensors, and bioelectrodes.

**Figure 1:**
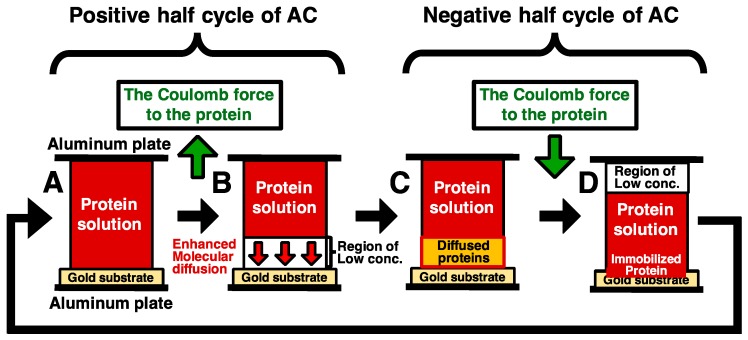
A model of immobilization method combining electrostatic transport and subsequent molecular diffusion of protein molecules (**A**). A pair of electrodes consist of a substrate electrode (the upper electrode) and the counter electrode (the lower electrode). The gap of the electrodes is filled with the protein solution, and low frequency AC electric field is applied. In this figure, green arrows show direction of Coulomb force (**B**). The proteins in solution are transported toward the upper electrode by Coulomb force with the application of positive half cycle of an AC electric field. The generated steep concentration gradient increases the transport rates of protein and thus enhances the molecular diffusion (**C**,**D**). When the electric field was sufficiently low during the gap between the positive and the negative half cycle, the proteins spread the low concentration area by enhanced molecular diffusion, followed by transportation of the proteins toward the substrate electrode by Coulomb force, and then the transported proteins are immobilized on a gold substrate surface. Repeating this cycle, the amount of immobilized proteins can be increased with the number of the repeated cycles.

We tried to solve this problem to enhance molecular diffusion by steep concentration gradient, which was generated by electrophoresis. The model is described below in detail (Figure 1). After spotting protein solution in a gap between a pair of electrodes, the proteins in solution are transported toward an electrode (the top electrode in Figure 1) by Coulomb force with the application of positive half cycle of an Alternating current (AC) electric field. (Figure 1A,B). During the application of positive half cycle of an AC electric field, the steep concentration gradient of proteins is generated by the formation of low concentration area in protein solution. The generated concentration gradient enhances the molecular diffusion (Figure 1B). When the electric field was sufficient low during the gap between the positive and the negative half cycle, the proteins spread the low concentration area by enhanced molecular diffusion, followed by transportation of the proteins toward an electrode (the down electrode in Figure 1) by Coulomb force, and then the transported proteins are immobilized on a gold substrate surface (Figure 1C,D). Repeating this cycle, the amount of immobilized proteins can be increased with number of the repeated cycles.

For sensor applications, we attempted to develop a new immobilization method permitting immobilization of both positively- and negatively-charged proteins and minimizing electrode reactions. To achieve these goals, we devised the new method based on the model described above. Because average voltage of AC is zero, electrode reactions and simple Coulomb force are cancelled during the one cycle. However, the steep gradient generated by electrophoresis seems to enhance molecule transportation. Binding through His-tag permits specific and highly-oriented immobilization of proteins, and these properties are advantageous for protein-based sensor applications. Therefore, we focused immobilization of His-tagged protein in this study. For evaluating immobilization efficiencies, the amounts of immobilized proteins were evaluated using His-tagged green fluorescent protein (GFP) and *Discosoma* sp. red fluorescent protein (DsRed) as model proteins. By applying a low-frequency AC electric field, we experimentally demonstrated that the His-tagged proteins were transported through molecular diffusion enhanced by generated steep concentration gradient, and that the immobilization of His-tagged proteins was strongly enhanced.

## 2. Materials and Methods

### 2.1. Materials

We purchased 3-Mercaptopropionic Acid (MPA) from Tokyo Chemical Industry (Tokyo, Japan). Nickel(II) Chloride Hexahydrate form Wako Pure Chemicals (Osaka, Japan). Chemically synthesized oligonucleotides primer was obtained from Japan Bio Services (Saitama, Japan). The restriction enzymes of *Bam*H I and *Hin*d III were purchased from TaKaRa (Tokyo, Japan). The PCR enzyme of Phusion^®^ High-Fidelity DNA polymerase was purchased from Thermo Fisher Scientific (Carlsbad, CA, USA). All other reagents were of analytical grade and were purchased from Sigma-Aldrich (St. Louis, MO, USA), Nacalai Tesque (Kyoto, Japan), or Wako Pure Chemicals Industries (Osaka, Japan).

### 2.2. Construction of His-Tagged DsRed Expression Vector

The plasmid of DsRed-Monomer was purchased from TaKaRa (Tokyo, Japan) The nucleotide sequence of DsRed gene was amplified by PCR with the following primer: 5′-CGC GGA TCC GCG ATG GAC AAC ACC GAG GAC GTC-3′, and reverse primer: 5′-CCC AAG CTT GGG CTG GGA GCC GGA GTG GCG-3′. The PCR product was digested with *Bam*H I and *Hin*d III and ligated into the pET21a vector digested with *Bam*H I and *Hin*d III (His-tagged DsRed-expression vector).

### 2.3. Purification of Reconbinant His-Tagged GFP and DsRed

The His-tag modified GFP expression vector (pGGFPH) was a kind gift from Professor H. Nakano (Nagoya University, Japan) [30]. His-tagged GFP and DsRed were overexpressed in *E. coli* Rosetta(DE3) (Novagen; Madison, WI, USA) and purified using Ni-NTA Superflow chromatography (Qiagen; Germantown, MD, USA) at 4 °C according to the manufacturer’s protocol, and then removed imidazole in the purified His-tagged GFP and DsRed using dialysis tubing (Spectrum Laboratories, Inc., Rancho Dominguez, CA, USA) according to the standard protocol for dialysis method [31]. The protein concentrations of purified His-tagged and DsRed were determined according to the method of Bradford using a protein assay kit (Bio-Rad; Benicia, CA, USA) with BSA as the standard.

For these prepared proteins, sodium dodecyl sulfate-polyacrylamide gel electrophoresis (SDS-PAGE) was performed with a 10% gel using the standard Laemmli method. Gel was run at a constant current of 20 mA per gel until the dye-front reached the bottom. A constant current of 20 mA was applied using a Bio-Rad powerpac 3000 power supply (Bio-Rad, Hercules, CA, USA). After electrophoresis, the gel was stained with Coomassie Brilliant Blue (CBB) R-250 (Wako, Osaka, Japan).

### 2.4. Preparation of the Modified Gold-Coated Substrate

A preparation method for protein immobilization substrate reported previously [27] was slightly modified as follows. Gold coverslips were prepared by attaching gold film (Nilaco, Tokyo, Japan) to glass coverslips (Matsunami, Tokyo, Japan) using adhesive tape (Nichiban, Tokyo, Japan). The gold coverslips were soaked in 1 N nitric acid for 1 h, and then rinsed with Milli-Q water. Next, using a 50 mL Falcon tube, both positive (gold coverslip) and negative (aluminum plate) electrodes were soaked in 10 mL of 1 M MPA solution dissolved in Milli-Q water. Electrodeposition of MPA was carried out at a constant direct current (DC) of 10 mA for 10 min at 37 °C with stirring. After the electrodeposition of MPA, the coverslips were then rinsed with Milli-Q water. To form a complex between Ni ions and the carboxyl groups of MPA bound to the coverslip, the MPA electrodeposited coverslips was modified by soaking in 150 mM Nickel Chloride solution of 10 mL for overnight at 37 °C with stirring and then rinsed with Milli-Q water.

### 2.5. Immobilization of His-Tagged GFP and DsRed with the Application of Low-Frequency AC Electric Field

**Figure 2:**
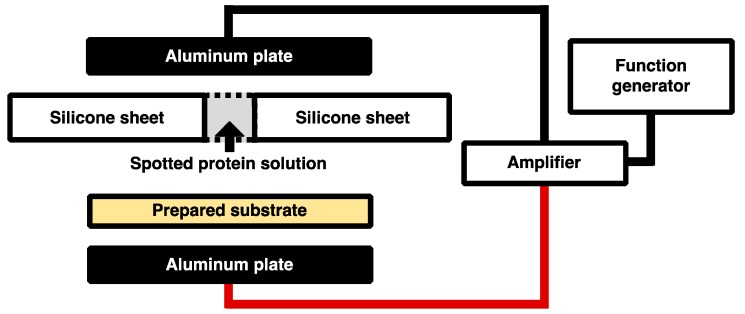
Overview of the experimental system. The AC electric field was generated by an amplifier with a function generator.

The concentrations of His-tagged GFP and DsRed solutions were adjusted to 70 μg/mL with Buffer A of 10 mM HEPES-NaOH pH 8.0, 1 mM dithiothreitol (DTT). Because ions in the solution decrease Coulomb force due to Debye screening, Buffer A contained only a minimal buffer 10 mM HEPES-NaOH pH 8.0 and anti-oxidant 1mM DTT. A total of 350 ng of His-tagged GFP and DsRed were spotted on the Ni-MPA-gold substrates covered silicone sheet with the holes of diameter 2 mm. After spotting of the solutions, the substrates with the silicone sheet were sandwiched between a pair electrodes of aluminum plates. The low frequency alternating current (AC) electric field was generated by the amplifier (4005 High Speed Power Amplifire, NF Electronic Instruments, Kanagawa, Japan) with the function generator (33210A, Agilent, Santa Clara, CA, USA) as shown in Figure 2. The conditions of applying electric field are described as follows: 10, 20, 40, 60, and 80 Hz at the constant applied voltage of 80 Vpp for the duration time of 30 min and 0, 20, 40, 60, 80, 100 and 120 Vpp of 20 Hz for the duration time of 30 min. After the application of electric field, the substrates were rinsed with Milli-Q water to remove unbound His-tagged GFP and DsRed and incubated at 37 °C for 1 h while shielded from light.

### 2.6. Immobilization of His-Tagged GFP with Time-Dependent Applied in Low-Frequency AC Electric Field

The concentration of His-tagged GFP solution was adjusted to 70 μg/mL with buffer A, and 350 ng of His-tagged GFP was spotted on the Ni-MPA-gold substrates covered silicone sheet with the holes of diameter 2 mm. After spotting of the solutions, the substrates with the silicone sheet were sandwiched between a pair electrodes of aluminum plates. The low frequency AC electric field was generated by the amplifier with the function generator as shown in Figure 2. The conditions of applying electric field are described as follows: 20 Hz at the constant applied voltage of 80 Vpp for the duration time of 10, 20, and 30 min. After the application of electric field, the substrate was rinsed with Milli-Q water to remove unbound His-tagged GFP and incubated at 37 °C for 1 h while shielded from light.

### 2.7. Immobilization of His-Tagged GFP Under Several pH Conditions

The concentrations of His-tagged GFP at a pH 5.0, 5.6, and 8.0 were adjusted to 70 μg/mL. The sample of pH 8.0 was prepared with Buffer A as described previously. The sample of pH 5.0 and pH 5.6 were prepared with Buffer B of 15 mM acetate buffer pH 5.0, 6.5 mM 2-Mercaptoethanol and Buffer C of 10 mM Tris-acetate buffer pH 5.6, 10 mM 2-Mercaptoethanol, respectively. Those samples containing 350 ng of His-tagged GFP were spotted on the Ni-MPA-gold substrates covered silicone sheet with the holes of diameter 2 mm. After spotting of the solutions, the substrates with the silicone sheet were sandwiched between a pair electrodes of aluminum plates. The low frequency AC electric field was generated by the amplifier with the function generator as shown in Figure 2. To prevent an aggregation of His-tagged GFP due to the acidic condition, the duration time of the application of electric field was shortened in this experiment. The conditions of applying electric field are described as follows: 20 Hz at the constant applied voltage of 80 Vpp for the duration time of 10 min. After the application of electric field, the substrate was rinsed with wash buffer (10 mM phosphate buffer pH 8.0 and 0.05% Tween 20) to remove non-specific absorption of His-tagged GFP and submitted to the determination of immobilized proteins. 

### 2.8. Observation and Determination of His-Tagged GFP and DsRed Immobilized on the Substrate Surfaces

The GFP and DsRed immobilized on the Ni-MPA-gold substrates were observed under a fluorescence microscope (Nikon ECLIPSE TE2000-U) equipped with 4 × 0.13 numerical aperture (NA) objective lenses. The immobilized His-tagged GFP (Excitation: 475 nm; Emission: 509 nm) was observed with a B-2A excitation wavelength (EX470/40, DM505, and EM520; Nikon) and the immobilized His-tagged DsRed (Excitation: 556 nm; Emission: 586 nm) was observed with a G-2A excitation wavelength (EX535/50, DM575, and EM590; Nikon). Fluorescent images were captured with a digital camera (Nicon D80), and the amount of immobilized His-tagged GFP and DsRed was estimated by analyzing the fluorescence intensity using Image J free software (NIH; http://rsbweb.nih.gov/ij/). The calibration line was obtained by polynomial fitting of the fluorescence intensities of standard samples. The scale was determined by comparison with the appropriate scale on the captured images. After the observation, the substrates were soaked in 0.5 M imidazole solution to dissociate the immobilized His-tagged GFP and DsRed and incubated at 37 °C overnight with stirring while shielded from light. The imidazole-treated substrates were observed under the fluorescence microscope and the images were captured with the digital camera.

## 3. Results and Discussion

### 3.1. Immobilization of His-Tagged GFP and DsRed with the Application of Low-Frequency AC Electric Field

**Figure 3:**
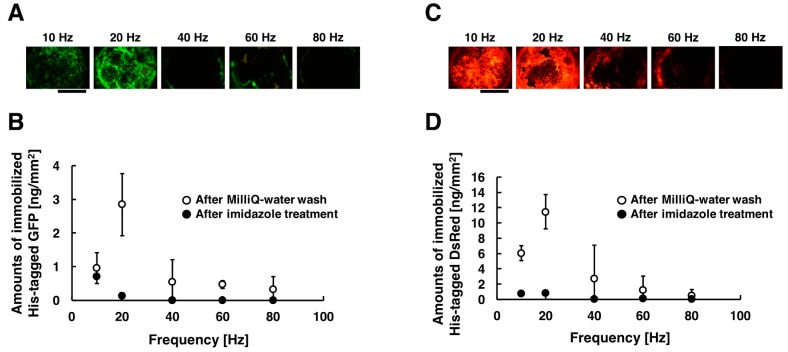
Fluorescent images and amounts of immobilized His-tagged GFP and DsRed by applying AC electric field. The conditions of applying AC electric field are described as follows: 10, 20, 40, 60, and 80 Hz at the constant applied voltage of 80 Vpp for the duration time of 30 min. (**A**) After the application of AC electric field, fluorescence images of immobilized His-tagged GFP. (**B**) After Milli-Q water wash and imidazole treatment, amounts of immobilized His-tagged GFP. (**C**) After the application of AC electric field, fluorescence images of immobilized His-tagged DsRed. (**D**) After Milli-Q water wash and imidazole treatment, amounts of immobilized His-tagged DsRed. Open and closed circle denote the amounts of immobilized His-tagged GFP and DsRed after Milli-Q water wash and imidazole treatment, respectively. Error bars represent the standard deviations from three independent experiments. Scale bar = 1 mm.

His-tagged GFP and DsRed were immobilized on the substrate by the method combining electrostatic transport and subsequent molecular diffusion. Figure 3A,C shows the acquired fluorescent images of the substrate when different frequencies were applied, and the amounts of immobilized His-tagged GFP and DsRed are summarized in Figure 3B,D. These experiments were carried out under the following conditions: 10, 20, 40, 60, and 80 Hz at the constant applied voltage of 80 Vpp for the duration of 30 min. The fluorescence intensities of the immobilized His-tagged GFP and DsRed had a peak at frequency of 20 Hz. On the other hands, the fluorescence intensities decreased with increasing frequency more than 40 Hz. Comparing the fluorescence intensities, His-tagged GFP and DsRed were immobilized more efficiently in the frequency of 20 Hz than that of 10, 40, 60, and 80 Hz. In our immobilization method, His-tagged GFP and DsRed were transported toward the substrate surfaces only once per one cycle of AC electric field. Under conditions of lower frequency (below 10 Hz), the amounts of immobilized His-tagged GFP and DsRed were thus limited by the number of cycles of AC electric field. On the other hand, under conditions of higher frequency (more than 40 Hz), time for molecular diffusion of proteins is insufficient because of the rapid change of electric field direction, and thus the amounts of immobilized His-tagged GFP and DsRed were significantly decreased. For the above reasons, under conditions of frequency of 20 Hz, the generated steep concentration gradient enhanced the molecular diffusion of His-tagged GFP and DsRed, and thus the immobilization of His-tagged GFP and DsRed were strongly enhanced. However, the amounts of immobilized His-tagged GFP was sufficiently less than that of immobilized His-tagged DsRed. This result suggests that the immobilization efficiencies of this method depend on properties of proteins such as the local structure beside the ligand, different folding structure, or differences in primary structure. In addition, almost all immobilized His-tagged GFP and DsRed were removed by washing the substrates with imidazole solution. This means that the immobilization of His-tagged GFP and DsRed were due to a complex formation between the His-tag and Ni ions on the substrate surfaces. 

**Figure 4:**
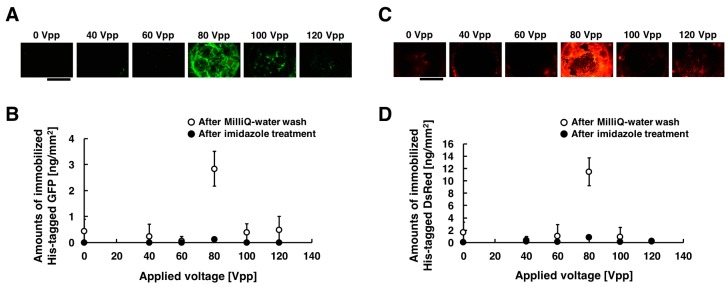
Fluorescent images and amounts of immobilized His-tagged GFP and DsRed by applying AC electric field. The conditions of applying AC electric field are described as follows: 0, 20, 40, 60, 80, 100, and 120 Vpp of 20 Hz for the duration time of 30 min. (**A**) After the application of AC electric field, fluorescence images of immobilized His-tagged GFP. (**B**) After Milli-Q water wash and imidazole treatment, amounts of immobilized His-tagged GFP. (**C**) After the application of AC electric field, fluorescence images of immobilized His-tagged DsRed. (**D**) After Milli-Q water wash and imidazole treatment, amounts of immobilized His-tagged DsRed. Open and closed circle denote the amounts of immobilized His-tagged GFP and DsRed after Milli-Q water wash and imidazole treatment, respectively. Error bars represent the standard deviations from three independent experiments. Scale bar = 1 mm.

Figure 4A,C show the acquired fluorescent images of the substrate when different voltages were applied, and the amounts of immobilized His-tagged GFP and DsRed are summarized in Figure 4B,D. These experiments were carried out under the following conditions: 20, 40, 60, 80, 100, and 120 Vpp of 20 Hz for duration of 30 min. In those figures, 0 Vpp of the applied voltage corresponds negative control experiment without voltage applications. The fluorescence intensities of the immobilized His-tagged GFP and DsRed had a peak at applied voltage of 80 Vpp, whereas it significantly decreased for the applied voltages of 20, 40, 60, 100, and 120 Vpp. Comparing the fluorescence intensities, His-tagged GFP and DsRed were immobilized more efficiently in the applied voltage of 80 Vpp than that of 20, 40, 60, 100, and 120 Vpp. When 100 and 120 Vpp AC voltages were applied to the substrate, the damage of the substrate surface was observed. Thus, it is attributable to the dissociation of the Ni-MPA complex modified on gold substrate surfaces that both His-tagged GFP and DsRed were not immobilized on the substrate. This is because the bubbles produced by excess AC voltage is also more likely to induce adverse effects as damaging on the prepared substrate surfaces. To evaluate the effect for heating of the device, the temperature of the chamber in the apparatus was measured by a non-contact thermometer (Thermo Phrase MT-500, NISSEI, Gunma, Japan). The chamber temperature raised from 20.6 °C to 22.4 °C during the application of 80 Vpp AC, indicating that an effect of Joule heating was sufficient low in our experiment. From these results, we determined that the immobilization of the maximum amounts can be obtained by the application of the voltage of 80 Vpp, 20 Hz both for His-tagged GFP and DsRed. The amounts of the immobilized His-tagged GFP and DsRed were approximately seven-fold higher than that in the absence of the application of low-frequency AC electric field (2.8 vs. 0.4 ng/mm^2^ for GFP, 12 vs. 1.7 ng/mm^2^ for DsRed). This is because the molecular weight of His-tagged GFP (approximately 27 kilo-Dalton) is almost the same as that of His-tagged DsRed (approximately 28 kilo-Dalton), and thus this means that the diffusion coefficient of His-tagged GFP is almost the same as that of His-tagged DsRed.

**Figure 5:**
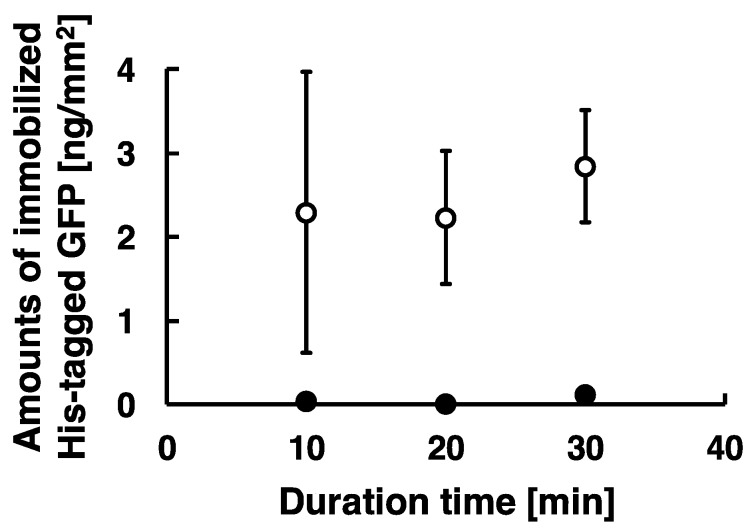
Scatter plots of amounts of immobilized His-tagged GFP by applying AC electric field. The conditions of applying AC electric field are described as follows: 20 Hz at the constant applied voltage of 80 Vpp for the duration time of 10, 20, and 30 min. Open circle and closed circle denote the amounts of immobilized His-tagged GFP after Milli-Q water wash and imidazole treatment. Error bars represent the standard deviations from three independent experiments.

To investigate effects of duration of low-frequency AC electric field on the amounts of immobilized proteins, we captured fluorescent images of immobilized His-tagged GFP of different duration time. The amounts of the immobilized proteins were analyzed as described previously and summarized as shown in Figure 5. These experiments were carried out under the following conditions: 20 Hz at the applied voltage of 80 Vpp for the duration time of 10, 20, and 30 min. The amounts of the immobilized His-tagged GFP for the duration time of 10, 20, and 30 min were approximately 2.3, 2.2, and 2.8 ng/mm^2^, respectively. The amounts of the immobilized His-tagged GFP was stably obtained for the duration time more than 20 min, whereas it was not stably obtained because of large error bars for the duration time of 10 min. These proteins were immobilized on the substrate by the method combining electrostatic transport and subsequent molecular diffusion. However, our results show the stabilization of immobilized protein required duration time above 20 min, and the amount of immobilized protein for duration of 10 min demonstrated much higher standard deviation. This means that the duration of 10 min was not enough for the transportation for immobilization of proteins because of limitations in the number of cycles of AC electric field, and our results strongly suggest that the amounts of the immobilized proteins depended on the length of duration time of low-frequency AC electric field (Figure 5). Therefore, stable immobilization of His-tagged GFP required at least a 20 min duration.

### 3.2. Immobilization of His-Tagged GFP under Several pH Conditions

**Figure 6:**
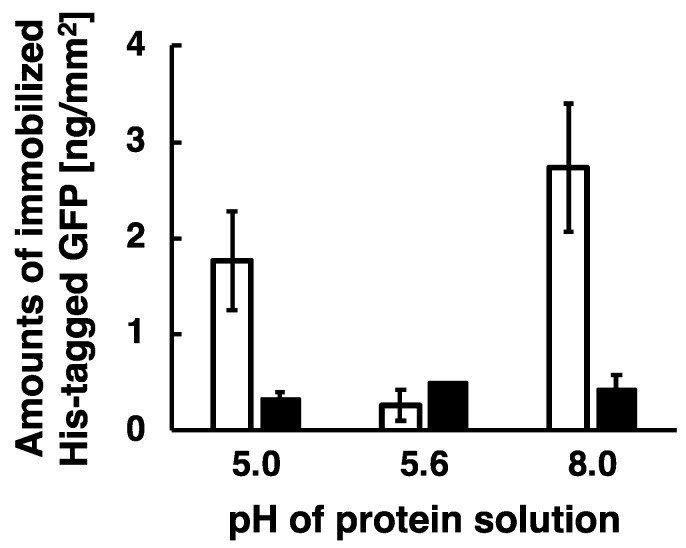
Histogram of amounts of immobilized His-tagged GFP at a pH 5.0, 5.6, and 8.0. To prevent an aggregation of His-tagged GFP due to the acidic condition, this experiment shortened the duration time of the application of AC electric field. The conditions of applying AC electric field are described as follows: 20 Hz at the constant applied voltage of 80 Vpp for the duration time of 10 min. White bars and black bars indicate the amounts of His-tagged GFP immobilized on the substrate surfaces after wash buffer and imidazole treatment, respectively. Error bars represent the standard deviations from three independent experiments.

To verify our model, the immobilization experiments were carried out under several pH conditions such as pH 5.0, 5.6, and 8.0. His-tag-Nickel bond is functional under this pH range as shown in previous report [32]. In the above experiments, His-tagged GFP and DsRed at a pH 8.0 were immobilized by the application of low-frequency AC electric field. GFP and DsRed has an isoelectric point of pH 5.6 and 4.2, respectively, and thus negatively charged His-tagged GFP and DsRed at a pH 8.0 were transported and immobilized by combining Coulomb force and subsequent generated steep concentration gradient. Here, we investigated effects of pH on immobilization efficiency around the isoelectric point (pI). The captured fluorescent images of the immobilized His-tagged GFP at a pH 5.0, 5.6, and 8.0 were analyzed to measure the amount of the immobilized proteins, and summarized as shown in Figure 6. The experiments were carried out only for His-tagged GFP. Because it is necessary to shift the pH below 4.0 for its charge to be positive, DsRed is more likely aggregated under such acidic conditions.

Applying low-frequency AC electric field, both the positively charged His-tagged GFP at a pH 5.0 and the negatively charged His-tagged GFP at a pH 8.0 were efficiently immobilized on the substrate surfaces, whereas the non-charged His-tagged GFP at a pH 5.6 (pI of the GFP) was not immobilized on the substrate surfaces. The amounts of the immobilized His-tagged GFP were 1.8 ng/mm^2^ for positive charge at a pH 5.0, 2.7 ng/mm^2^ for negative charge at a pH 8.0, and 0.3 ng/mm^2^ for non-charge at a pH 5.6. This is because electrically charged proteins were transported by applying low-frequency AC electric field and then the generated steep concentration gradient of proteins enhanced molecular diffusion, resulting in the electrically charged proteins being immobilized by this method. Therefore, we experimentally demonstrated that the immobilization of proteins by the application of low-frequency AC electric field was enhanced as our model proposed. However, the amount of immobilized His-tagged GFP was not stably obtained because of the duration time of 10 min as described above. As a result, almost all the immobilized His-tagged GFP were removed by washing the substrates with imidazole solution, and therefore we demonstrate that His-tagged GFP were immobilized by the formation of a complex between the His-tag and Ni ions on the substrate surfaces. Auer et al. reported that Co.(III)-NTA modified solid was suitable for tight immobilization of His-tagged protein in comparison to Ni(II)-NTA modified solid [33]. For future research, we will apply this immobilization method to Co.(III)-NTA modified solid for more stable and higher density immobilization of His-tagged proteins.

## 4. Conclusions

In this study, we developed a new immobilization method by combining electrostatic transport and subsequent molecular diffusion enhanced by steep concentration gradient of protein molecules. Our developed immobilization method is based on a model that proteins transport toward substrate surface due to steep concentration gradient generated by low-frequency AC electric field. The immobilization of the maximum amounts can be obtained by the application of the voltage of 80 Vpp, 20 Hz both for His-tagged GFP and DsRed. The amounts of the immobilized His-tagged GFP and DsRed were approximately seven-fold higher than those in the absence of the application of low-frequency AC electric field. In addition, the positively and negatively charged protein at both acidic and alkaline pH were immobilized by applying low-frequency AC electric field, whereas the non-charged His-tagged protein at the pH with isoelectric points of zero was not immobilized. Therefore, unless the pH is equal to pI, we experimentally demonstrate that the immobilization of electrically charged proteins was strongly enhanced by the method combining electrostatic transport and subsequent molecular diffusion. These results strongly suggest the enhancement of protein immobilization took place according to our model. In the future, our developed immobilization method will be a key technique in the production of protein arrays, biosensors, and bioelectrodes for use in the analytical and bioelectronics fields.

## Abbreviations

MPA, 3-Mercaptopropionic Acid; SAM, self-assembled monolayer; NTA, nitrilotriacetic acid; His, Histidine; AC, Alternating current; GFP, green fluorescent protein; DsRed, *Discosoma sp.* red fluorescent protein; DTT, dithiothreitol; HEPES, 4-(2-hydroxyethyl)-1-piperazineethanesulfonic acid; BSA, bovine serum albumin; PCR, polymerase chain reaction; pI; isoelectric point.

## References

[1] Bonroy K., Frederix F., Reekmans G., Dewolf E., De Palma R., Borghs G., Declerck P., Goddeeris B. (2006). Comparison of random and oriented immobilisation of antibody fragments on mixed self-assembled monolayers. J. Immunol. Methods.

[2] Pei Z., Anderson H., Myrskog A., Dunér G., Ingemarsson B., Aastrup T. (2010). Optimizing immobilization on two-dimensional carboxyl surface: PH dependence of antibody orientation and antigen binding capacity. Anal. Biochem..

[3] Rusmini F., Zhong Z., Feijen J. (2007). Protein Immobilization Strategies for Protein Biochips. Biomacromolecules.

[4] Ha T.H., Jung S.O., Lee J.M., Lee K.Y., Lee Y., Park J.S., Chung B.H. (2007). Oriented immobilization of antibodies with GST-fused multiple Fc-specific B-domains on a gold surface. Anal. Chem..

[5] Lee J.M., Park H.K., Jung Y., Kim J.K., Jung S.O., Chung B.H. (2007). Direct immobilization of protein G variants with various numbers of cysteine residues on a gold surface. Anal. Chem..

[6] Pyun J.C., Kim S.D., Chung J.W. (2005). New immobilization method for immunoaffinity biosensors by using thiolated proteins. Anal. Chem..

[7] Ge H. (2000). UPA, a Universal Protein Array System for Quantitative Detection of Protein-Protein, Protein-DNA, Protein-RNA and Protein-Ligand Interactions. Nucleic Acid Res..

[8] Holt L.J., Büssow K., Walter G., Tomlinson I.M. (2000). By-passing selection: Direct screening for antibody–antigen interactions using protein arrays. Nucleic Acids Res..

[9] Pollak A., Blumenfeld H., Wax M., Baughn R.L., Whitesides G.M. (1980). Enzyme immobilization by condensation copolymerization into crosslinked polyacrylamide gels. J. Am. Chem. Soc..

[10] Kim D., Karns K., Tia S.Q., He M., Herr A.E. (2012). Electrostatic protein immobilization using charged polyacrylamide gels and cationic detergent microfluidic western blotting. Anal. Chem..

[11] Gao Y., Kyratzis I. (2008). Covalent immobilization of proteins on carbon nanotubes using the cross-linker 1-ethyl-3-(3-dimethylaminopropyl) carbodiimide—A critical assessment. Bioconjug. Chem..

[12] MacBeath G., Schreiber S.L. (2000). Printing proteins as microarrays for high-throughput function determination. Science.

[13] Camarero J.A. (2008). Recent developments in the site-specific immobilization of proteins onto solid supports. Pept. Sci..

[14] Gauthier M.A., Klok H.A. (2008). Peptide/protein–polymer conjugates: Synthetic strategies and design concepts. Chem. Commun..

[15] Frasconi M., Mazzei F., Ferri T. (2010). Protein immobilization at gold–thiol surfaces and potential for biosensing. Anal. Bioanal. Chem..

[16] Chen H., Lee M., Choi S., Kim J.-H., Choi H.-J., Kim S.-H., Lee J., Koh K. (2007). Comparative Study of Protein Immobilization Properties on Calixarene Monolayers. Sensors.

[17] Sigal G.B., Bamdad C., Barberis A., Strominger J., Whitesides G.M. (1996). A self-assembled monolayer for the binding and study of histidine-tagged proteins by surface plasmon resonance. Anal. Chem..

[18] Samanta D., Sarkar A. (2011). Immobilization of bio-macromolecules on self-assembled monolayers: Methods and sensor applications. Chem. Soc. Rev..

[19] Ertekin Ö., Öztürk S., Öztürk Z.Z. (2016). Label Free QCM Immunobiosensor for AFB1 Detection Using Monoclonal IgA Antibody as Recognition Element. Sensors.

[20] Miyao H., Ikeda Y., Shiraishi A., Kawakami Y., Sueda S. (2015). Immobilization of immunoglobulin-G-binding domain of protein A on a gold surface modified with biotin ligase. Anal. Biochem..

[21] Holmberg A., Blomstergren A., Nord O., Lukacs M., Lundeberg J., Uhlén M. (2005). The biotin-streptavidin interaction can be reversibly broken using water at elevated temperatures. Electrophoresis.

[22] Willard F.S., Siderovski D.P. (2006). Covalent immobilization of histidine-tagged proteins for surface plasmon resonance. Anal. Chem..

[23] Khan F., He M., Taussig M.J. (2006). Double-hexahistidine tag with high-affinity binding for protein immobilization, purification, and detection on Ni−nitrilotriacetic acid surfaces. Anal. Chem..

[24] Li X., Song S., Pei Y., Dong H., Aastrup T., Pei Z. (2016). Oriented and reversible immobilization of His-tagged proteins on two-and three-dimensional surfaces for study of protein–protein interactions by a QCM biosensor. Sens. Actuators B Chem..

[25] Lesaicherre M.L., Lue R.Y., Chen G.Y., Zhu Q., Yao S.Q. (2002). Intein-mediated biotinylation of proteins and its application in a protein microarray. J. Am. Chem. Soc..

[26] Girish A., Sun H., Yeo D.S., Chen G.Y., Chua T.K., Yao S.Q. (2005). Site-specific immobilization of proteins in a microarray using intein-mediated protein splicing. Bioorg. Med. Chem. Lett..

[27] Oshige M., Yumoto K., Miyata H., Takahashi S., Nakada M., Ito K., Tamegai M., Kawaura H., Katsura S. (2013). Immobilization of His-tagged proteins on various solid surfaces using NTA-modified chitosan. Open J. Polym. Chem..

[28] Miyata H., Yumoto K., Itoh K., Sasahara M., Kawaura H., Oshima N., Suzuki T., Takahashi S., Oshige M., Katsura S. (2014). Immobilization of His-Tagged Proteins through Interaction with L-Cysteine Electrodeposited on Modified Gold Surfaces. Key Eng. Mater..

[29] Hochuli E., Bannwarth W., Döbeli H., Gentz R., Stüber D. (1988). Genetic approach to facilitate purification of recombinant proteins with a novel metal chelate adsorbent. Nat. Biotechnol..

[30] Kinpara T., Mizuno R., Murakami Y., Kobayashi M., Yamaura S., Hasan Q., Morita Y., Nakano H., Yamane T., Tamiya E. (2004). A picoliter chamber array for cell-free protein synthesis. J. Biochem..

[31] Sambrook J., Fritsch E.F., Maniatis T. (1989). Molecular Cloning: A Laboratory Manual.

[32] Chaga G.S. (2001). Twenty-five years of immobilized metal ion affinity chromatography: Past, present and future. J. Biochem. Biophys. Methods.

[33] Auer S., Azizi L., Faschinger F., Blazevic V., Vesikari T., Gruber H.J., Hytönen V.P. (2017). Stable immobilisation of His-tagged proteins on BLI biosensor surface using cobalt. Sens. Actuators B Chem..

